# Analyzing the correlation between acute ischemic stroke and triglyceride-glucose index based on ordered logistic regression

**DOI:** 10.3389/fneur.2025.1500572

**Published:** 2025-02-05

**Authors:** Liu He, Rong Li, Lei Wang, Xi Zhu, Qiang Zhou, Zhiyong Yang, Hua Liu

**Affiliations:** Department of Neurology, The Affiliated Hospital of Southwest Jiaotong University, The Third People's Hospital of Chengdu, Chengdu, Sichuan, China

**Keywords:** acute ischemic stroke, triglyceride-glucose index, insulin resistance, intravenous thrombolysis, modified Rankin scale

## Abstract

**Objective:**

To investigate the association between insulin resistance, measured by the triglyceride-glucose (TyG) index, and clinical outcomes in patients with acute ischemic stroke who underwent intravenous thrombolysis with alteplase.

**Methods:**

This retrospective study included 165 patients with acute ischemic stroke treated with intravenous alteplase. Insulin resistance was evaluated using the TyG index, and its relationship with the modified Rankin Scale (mRS) scores was analyzed. The analysis was conducted using R software (version R 4.1.3) to evaluate the correlation between the TyG index and functional outcomes at 14, 30, and 90 days post-stroke.

**Results:**

The study found that each unit increase in the TyG index significantly raised the risk of poor functional outcomes at 14 days (OR 9.86; 95% CI: 3.32–32.21; *P* < 0.001), 30 days (OR 5.82; 95% CI: 2.08–17.45; *P* = 0.001), and 90 days (OR 9.79; 95% CI: 3.33–31.66; *P* < 0.001) following a stroke. Higher TyG index values were associated with worse neurological outcomes. Although male gender, older age, and smoking were also linked to poorer outcomes, these associations did not reach statistical significance.

**Conclusion:**

The findings suggest that a higher TyG index, indicating greater insulin resistance, is associated with worse neurological outcomes in stroke patients. Early intervention targeting insulin resistance may improve clinical outcomes in ischemic stroke patients, and further research is needed to explore additional factors affecting neurological recovery.

## 1 Introduction

Stroke is a major cause of death and disability worldwide, significantly straining healthcare systems and impacting society. Acute ischemic stroke is the most prevalent type, representing 69.6% to 72.8% of new stroke cases in China (1, 2). Recent data indicate that the in-hospital mortality rate for acute ischemic stroke patients in China, with a median hospital stay of 11 days, is 0.5%. The mortality rate increases from 1.5% to 3.2% at 3 months post-stroke and from 3.4% to 6.0% at 1 year (4–6). The disability rate after 3 months of illness is from 14.6% to 23.1%, and the disability rate after 1 year is from 13.9% to 14.2% (4, 5, 7). The study of stroke risk factors is important for the early detection of high-risk populations and improving their outcomes. Insulin resistance (insulin resistance, IR) is prevalent in patients with ischemic stroke (IS). Previous research have demonstrated that insulin resistance is independently linked to adverse clinical consequences of ischemic stroke, exacerbating neurological deterioration during hospitalization and triggering recurrent (3) of ischemic stroke. IR is regarded as a major risk factor for the development of stroke.

Insulin resistance (IR) is the main feature of the metabolic syndrome (8). Meta-analysis showed that high IR levels are associated with (9) risk of poor functional prognosis and increased risk of neurological deterioration in acute stroke patients. The triacylglycerol-glucose index (TyG index) is a simple and reliable indicator used as a surrogate for insulin resistance (10). High TyG index is linked to new IS in the general population (11, 12). Compared to IS patients with a low TyG index, those with a high TyG index have an elevated risk of stroke recurrence and increased mortality in Yang et al. (12), Triacylglycerol (triglyceride, TG) and fasting blood glucose (fasting blood glucose, FBG) as an element of the formula for calculating the TyG index, Early neurological deterioration (early neurological deterioration, END) of the known predictors of Martin and Price (13) Yu et al. (14). The relationship between TyG index and immediate term neurological recovery and prognosis in IS patients is unclear. Therefore, this study mainly analyzes the association between TyG index and the recovery and long-term neurological function after the occurrence of acute ischemic stroke. It aims to offer valuable insights for clinical research on ischemic stroke.

## 2 Materials and methods

### 2.1 Inclusion and exclusion criteria

This study retrospectively included 165 patients with actue ischemic stroke patients treated with intravenous alteplase, who completed Triglyceride Glucose (TyG) Index and completed the Modified Rankin scale (mRs) at a tertiary hospital in Chengdu between 01.2023 to 12.2023. The study received approval from the Hospital's Ethics Committee under approval number 2023-S-56 and followed the principles of the Declaration of Helsinki. Written informed consent was obtained from all participants prior to the commencement of the study.

The inclusion criteria for patients were: (1) meeting the diagnostic criteria for acute ischemic stroke as defined in the 2014 Chinese Guidelines for the Diagnosis and Treatment of Acute Ischemic Stroke, confirmed by head CT or MRI; (2) first episode of stroke; (3) time from onset to admission < 4.5 h and receiving intravenous alteplase thrombolysis treatment; and (4) age ≥ 18 years.

**Figure 1:**
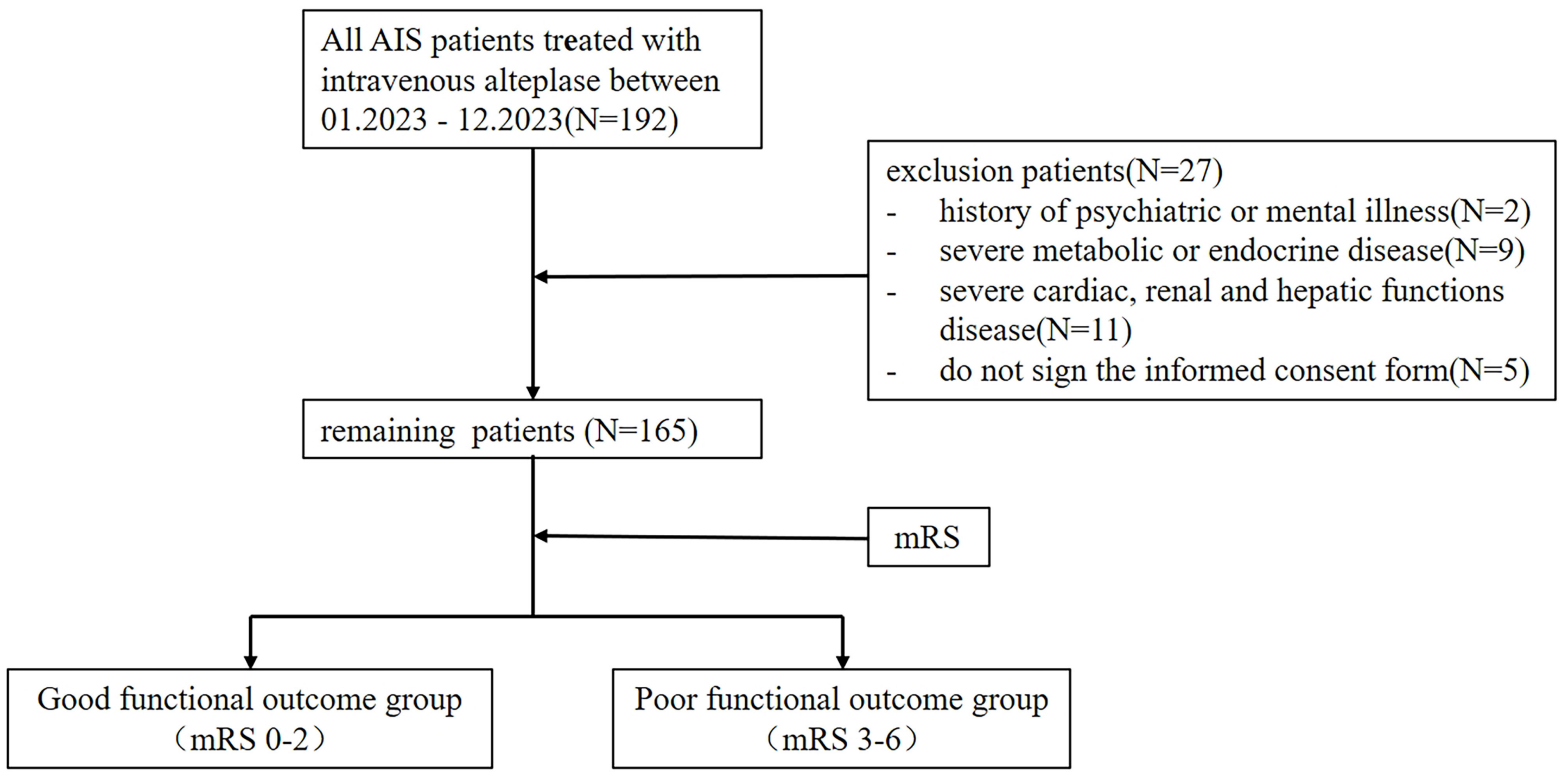
The procedure of the study.

The exclusion criteria were: (1) history of psychiatric or mental illness; (2) severe metabolic or endocrine diseases; (3) severe cardiac, renal, or hepatic dysfunction; and (4) patients or their families not signing the informed consent form. The patient selection process was depicted in Figure 1.

#### 2.1.1 Data collection

##### 2.1.1.1 Clinical baseline data

Clinical baseline data were collected from the hospital's medical record system, including sex, age, initial systolic and diastolic blood pressure on admission, smoking status (defined as smoking at least one cigarette per day on average in the past year), alcohol consumption (defined as daily intake ≥100 ml with alcohol content ≥50% for at least 1 year), medical history (e.g., hypertension, diabetes, dyslipidemia, stroke, coronary artery disease, and atrial fibrillation), medication history (e.g., antiplatelet agents, anticoagulants, antihypertensive drugs, statins, and hypoglycemic agents), pre-thrombolysis modified Rankin Scale (mRS) scores, and door-to-needle (DNT) times for intravenous thrombolysis. Follow-up mRS assessments were performed at 14, 30, and 90 days post-onset.

##### 2.1.1.2 Laboratory data

Laboratory data included platelet count, international normalized ratio (INR), fibrinogen (Fg), high-density lipoprotein (HDL), low-density lipoprotein (LDL), fasting triglycerides (TG), and fasting glucose (GLU). Venous blood samples were collected and analyzed by the hospital's laboratory department using the following methods:

Platelet count was measured using a 2 ml venous blood sample treated with EDTA anticoagulant (concentration: 1.5–2.2 mg/ml blood) and analyzed by semiconductor laser-based flow cytometry with the Mindray BC-7500 automated five-part differential hematology analyzer. International Normalized Ratio (INR) was determined from a 2.7 ml venous blood sample containing 0.109 M (3.2%) sodium citrate as an anticoagulant, using the STA R fully automated coagulation analyzer (Diagnostica Stago). Fibrinogen (Fg) levels were also assessed from the same blood specimen containing sodium citrate using the STA R analyzer. High-Density Lipoprotein Cholesterol (HDL-C) and Low-Density Lipoprotein Cholesterol (LDL-C) were measured from 3 ml serum using the Mindray BS-2800M fully automated biochemical analyzer, with HDL-C assessed by the masking method and LDL-C by the homogeneous enzymatic colorimetric method. Triglycerides (TG) were determined from 3 ml serum using the glycerol oxidase method on the same analyzer. Glucose (GLU) levels were assessed from 1–2 ml serum using an enzymatic method with the Mindray BS-2800M analyzer. The TyG index was calculated using the formula: ln [fasting triglyceride (mg/dL) × fasting blood glucose (mg/dL)/2] (15–22).

#### 2.1.2 Modified Rankin scale

The modified Rankin scale is designed to assess the degree of neurological recovery in individuals after stroke. Divided as a 0–6 level. Grade 0: Asymptomatic. Level 1: Despite symptoms, no significant disability, able to perform usual duties and activities. Level 2: Moderate disability, not able to complete all previously possible activities, but capable of managing personal matters independently. Level 3: moderate disability, needs some assistance, but capable of walking independently. Level 4: severely disabled, not able to walk independently, and incapable of managing personal care and physical needs. Grade 5: severe disability, bedridden, incontinence, continuous care, and care. Grade 6: death. For all patients, we evaluated their MRs scores before, 14 days, 30 days, and 90 days after Alteplase intravenous thrombolysis, respectively.

### 2.2 Sample size calculation

Logistic regression analysis was used. A minimum of 50 paired cases is required for logistic regression, and since the study divided patients into two groups based on prognosis, at least 100 cases were needed. For multivariate analysis, the total number of observations must exceed 100, with the sample size being 10–20 times the number of variables. This study, which aims to explore the correlation between the TyG index and prognosis in acute ischemic stroke and includes five covariates, required a minimum of 100 cases (23).

### 2.3 Statistical analysis

Data entry was carried out using Excel 2016, and after error verification, statistical analysis was carried out using R 4.1.3 software. Descriptive statistics were used to analyze the general information. Data with a normal distribution were reported as mean ± standard deviation (x ± s), whereas non-normally distributed data were presented as median values. Count data were presented as frequencies or composition ratios.

One-way analyses were conducted with analysis of variance (for normally distributed data) or the Kruskal-Wallis test (for non-normally distributed data). Frequency or proportion data were analyzed using the Chi-square test. Multifactorial analyses were conducted using a hierarchical logistic regression model with two levels. The relationships between independent and dependent variables were assessed through odds ratios (ORs), with 95% confidence intervals (CIs) providing the range for these OR values. The threshold for significance was set at α = 0.05, with differences deemed statistically significant if *P* < 0.05.

## 3 Results

### 3.1 The distribution characteristics of baseline information of questionnaire subjects

This analysis included 165 individuals who received intravenous alteplase thrombolysis. The study group included 90 males (54.55%) and 75 famales (45.45%). The mean age of the study population was 70.84 ± 12.15 years. The SBP before intravenous thrombolysis was 147.44 ± 24.16 mmHg, and the DBP was 85.75 ± 17.56 mmHg. Most of the study participants did not engage in smoking or alcohol consumption. The proportion of study with diabetes, stroke, coronary heart disease, atrial fibrillation and hyperlipidemia was also smaller than those with the previous history, while the opposite was true for hypertension. The proportion of study who previously took antiplatelet, anticoagulation, statins, and hypoglycemic drugs was also smaller than that of patients with the above history of medication, with an opposite history of antihypertensive drugs. Before thrombolysis MRs were 0 points:0(0%), 1 points:1 (6.76%), 2 points:30 (18.18%); 3 points:43 (26.06%); 4 points:66 (40.00%); 5 points:15 (9.09%); 6 points:0 (0.00%). Table 1 presents the patient characteristics in the study cohort.

**Table 1:** Overall patient baseline (*N* = 165).

**Variable**	**Number of cases/composition ratio**
**Sex**	
Male	90 (54.55%)
Female	75 (45.45%)
Age (years)	70.84 ± 12.15
First hospitalization SBP (mmHg)	147.44 ± 24.16
First hospitalization DBP (mmHg)	85.75 ± 17.56
**Smoking**	
No	92 (55.76%)
Yes	73 (44.24%)
**Alcohol consumption**	
No	115 (69.70%)
Yes	50 (30.30%)
**History of hypertension**	
No	60 (36.36%)
Yes	105 (63.64%)
**History of diabetes**	
No	125 (75.76%)
Yes	40 (24.24%)
**History of dyslipidemia**	
No	134 (81.21%)
Yes	31 (18.79%)
**History of cerebral infarction**	
No	137 (83.03%)
Yes	28 (16.97%)
**History of CHD**	
No	144 (87.27%)
Yes	21 (12.73%)
**History of atrial fibrillation**	
No	130 (78.79%)
Yes	35 (21.21%)
**History of antiplatelet drugs**	
No	125 (75.76%)
Yes	40 (24.24%)
**History of anticoagulant drugs**	
No	152 (92.12%)
Yes	13 (7.88%)
**History of antihypertensive drugs**	
No	69 (41.82%)
Yes	96 (58.18%)
**History of statin drugs**	
No	121 (73.33%)
Yes	44 (26.67%)
**History of hypoglycemic drugs**	
No	131 (79.39%)
Yes	34 (20.61%)
**mRS score before thrombolysis**	
0	0 (0.00%)
1	11 (6.67%)
2	30 (18.18%)
3	43 (26.06%)
4	66 (40.00%)
5	15 (9.09%)
6	0 (0.00%)

A descriptive analysis of the results of postadmission laboratory examinations of the study subjects as well as post-thrombolysis MRs. DNT (door to needle time) was 70.49 ± 38.83 mins. The median number of platelets was 178.80 ± 63.35 X109/L, and the median INR was 1.20 ± 0.41. The median value of the fibrinogen was 2.93 ± 1.35 g/L. The median value of the total cholesterol was 3.94 ± 1.06 mmol/L. The median value of the HDL was 1.24 ± 0.30 mmol/L. The median amount of LDL was 2.21 ± 0.83 mmol/L. The median total triglyceride was 1.17 ± 0.36 mmol/L. The median fasting level of blood glucose was 7.13 ± 2.17 mmol/L. The median of TyG index was 9.13 ± 0.62. The distribution of patient mRS at 14 days after thrombolysis was: 0 point: 34 (20.61%); 1 point: 43 (26.06%); 2 points: 25 (15.15%), 3 points: 9 (5.45%); 4 points: 23 (13.94%); 5 points: 20 (12.12%); 6 points: 11 (6.67%). The distribution of patient mRS at 30 days after thrombolysis was: 0 point: 38 (23.03%); 1 point: 41 (24.85%); 2 points: 19 (11.52%); 3 points: 14 (8.48%); 4 points: 21 (12.73%); 5 points: 16 (9.70%); 6 points: 16 (9.70%). The distribution of patient mRS scores at 90 days after thrombolysis was: 0 point: 45 (27.27%); 1 point: 39 (23.64%); 2 points: 10 (6.06%); 3 points: 24 (14.55%); 4 points: 16 (9.70%); 5 points: 11 (6.67%); 6 points: 20 (12.12%). Table 2 presents the detailed results.

**Table 2:** Descriptive statistics general population examination results (*N* = 165).

**Variables**	**Number of cases/composition ratio**
DNT (min)	70.49 ± 38.83
PLT (10^9^/L)	178.80 ± 63.35
INR	1.20 ± 0.41
Fg (g/L)	2.93 ± 1.35
TG (mmol/L)	3.94 ± 1.06
HDL (mmol/L)	1.24 ± 0.30
LDL (mmol/L)	2.21 ± 0.83
TyG index	9.13 ± 0.62
**14 d mRS**	
0	34 (20.61%)
1	43 (26.06%)
2	25 (15.15%)
3	9 (5.45%)
4	23 (13.94%)
5	20 (12.12%)
6	11 (6.67%)
**30 dmRS**	
0	38 (23.03%)
1	41 (24.85%)
2	19 (11.52%)
3	14 (8.48%)
4	21 (12.73%)
5	16 (9.70%)
6	16 (9.70%)
**90 dmRS**	
0	45 (27.27%)
1	39 (23.64%)
2	10 (6.06%)
3	24 (14.55%)
4	16 (9.70%)
5	11 (6.67%)
6	20 (12.12%)

Non-normal data, mean ± standard deviation of non-normal data expressed as mean.

### 3.2 Analysis of patients with different functional outcomes of the TyG index results at 14 days after intravenous thrombolysis

A normality test of the HyG index results revealed that the data were skewed. Consequently, the study utilized the Kruskal-Wallis test and Chi-square test (Table 3), one-way analysis of variance (ANOVA) (Table 4), and the median test for analysis.

**Table 3:** Analysis of baseline characteristics with different functional outcome groups at 14 days after intravenous thrombolysis.

**Variable**	**Good functional outcome (mRS 0–2, *N =* 102)**	**Poor functional outcome (mRS 3–6, *N =* 63)**	***P***
**Sex**			0.027
Male	63 (61.76%)	27 (42.86%)	
Female	39 (38.24%)	36 (57.14%)	
Age (years)	69.48 ± 11.52	73.03 ± 12.88	0.076
First hospitalization SBP (mmHg)	148.38 ± 24.58	145.74 ± 23.52	0.520
First hospitalization DBP (mmHg)	84.65 ± 17.18	87.75 ± 18.23	0.312
**Smoking**			0.277
No	53 (51.96%)	39 (61.90%)	
Yes	49 (48.04%)	24 (38.10%)	
**Alcohol consumption**			0.109
No	66 (64.71%)	49 (77.78%)	
Yes	36 (35.29%)	14 (22.22%)	
**History of hypertension**			0.596
No	35 (34.31%)	25 (39.68%)	
Yes	67 (65.69%)	38 (60.32%)	
**History of diabetes**			0.300
No	74 (72.55%)	51 (80.95%)	
Yes	28 (27.45%)	12 (19.05%)	
**History of dyslipidemia**			0.338
No	80 (78.43%)	54 (85.71%)	
Yes	22 (21.57%)	9 (14.29%)	
**History of cerebral infarction**			0.935
No	84 (82.35%)	53 (84.13%)	
Yes	18 (17.65%)	10 (15.87%)	
**History of CHD**			1.000
No	89 (87.25%)	55 (87.30%)	
Yes	13 (12.75%)	8 (12.70%)	
**History of atrial fibrillation**			1.000
No	80 (78.43%)	50 (79.37%)	
Yes	22 (21.57%)	13 (20.63%)	
**History of antiplatelet drugs**			0.507
No	75 (73.53%)	50 (79.37%)	
Yes	27 (26.47%)	13 (20.63%)	
**History of anticoagulant drugs**			0.246
No	96 (94.12%)	56 (88.89%)	
Yes	6 (5.88%)	7 (11.11%)	
No	41 (40.20%)	28 (44.44%)	
Yes	61 (59.80%)	35 (55.56%)	
**History of statin drugs**			0.638
No	73 (71.57%)	48 (76.19%)	
Yes	29 (28.43%)	15 (23.81%)	
**History of hypoglycemic drugs**			0.849
No	80 (78.43%)	51 (80.95%)	
Yes	22 (21.57%)	12 (19.05%)	

Continuous variables are expressed as Mean ± SD and categorical variables are expressed as No. (%).

**Table 4:** Analysis of laboratory test results with different functional outcome groups at 14 days after intravenous thrombolysis [mean ± SD/No. (%), *N* = 165].

**Variable**	**Good functional outcome (mRS 0–2, *N =* 102)**	**Poor functional outcome (mRS 3–6, *N =* 63)**	***P***
DNT (min)	70.58 ± 38.04	70.35 ± 40.39	0.971
PLT (10^9^/L)	170.47 ± 55.64	188.46 ± 70.52	0.123
INR	1.22 ± 0.49	1.16 ± 0.25	0.369
Fg (g/L)	2.83 ± 1.43	3.08 ± 1.25	0.339
TG (mmol/L)	3.97 ± 1.12	3.90 ± 0.94	0.675
HDL (mmol/L)	1.22 ± 0.30	1.26 ± 0.31	0.518
LDL (mmol/L)	2.23 ± 0.84	2.18 ± 0.82	0.751
TyG index	8.63 ± 0.30	8.85 ± 0.45	< 0.001

The investigators were divided into two groups based on functional outcomes: those with a good functional outcome (mRS 0–2) and those with a poor functional outcome (mRS 3–6). Fourteen days after intravenous thrombolysis, 102 people were included in the good functional prognosis group and 63 people in the poor functional prognosis group. Except for gender, there were no meaningful differences in the baseline characteristics of patients between the two outcome groups (*P* > 0.05). Table 3 summarizes the results.

There was no difference in DNT, PLT, INR, Fg, TG, HDL, and LDL between the two groups. The median TyG index in the good functional outcome group was 8.63 ± 0.30, and in the poor functional outcome group was 8.85 ± 0.45. Notable differences were detected between the two groups (*P* < 0.001). Findings of the analysis are presented in Table 4.

### 3.3 Analysis of patients with different functional outcomes of the TyG index results at 30 days after intravenous thrombolysis

Since the normality test revealed that the HyG index data were skewed, the analysis utilized the Kruskal-Wallis test and Chi-square test (Table 5), one-way analysis of variance (ANOVA) (Table 6), and the median test.

**Table 5:** Analysis of baseline characteristics with different functional outcome groups at 30 days after intravenous thrombolysis.

**Variable**	**Good functional outcome (mRS 0–2, *N =* 98)**	**Poor functional outcome (mRS 3–6, *N =* 67)**	***P***
**Sex**			0.054
Male	60 (61.22%)	30 (44.78%)	
Female	38 (38.78%)	37 (55.22%)	
Age	69.72 ± 11.70	72.46 ± 12.68	0.162
First hospitalization SBP(mmHg)	148.05 ± 24.94	146.44 ± 23.03	0.688
First hospitalization DBP(mmHg)	83.80 ± 17.12	88.89 ± 17.96	0.090
**Smoking**			0.316
No	51 (52.04%)	41 (61.19%)	
Yes	47 (47.96%)	26 (38.81%)	
**Alcohol consumption**			0.098
No	63 (64.29%)	52 (77.61%)	
Yes	35 (35.71%)	15 (22.39%)	
**History of hypertension**			1.000
No	36 (36.73%)	24 (35.82%)	
Yes	62 (63.27%)	43 (64.18%)	
**History of diabetes**			0.519
No	72 (73.47%)	53 (79.10%)	
Yes	26 (26.53%)	14 (20.90%)	
**History of dyslipidemia**			0.659
No	78 (79.59%)	56 (83.58%)	
Yes	20 (20.41%)	11 (16.42%)	
**History of cerebral infarction**			0.956
No	82 (83.67%)	55 (82.09%)	
Yes	16 (16.33%)	12 (17.91%)	
**History of CHD**			0.625
No	84 (85.71%)	60 (89.55%)	
Yes	14 (14.29%)	7 (10.45%)	
**History of atrial fibrillation**			1.000
No	77 (78.57%)	53 (79.10%)	
Yes	21 (21.43%)	14 (20.90%)	
**History of antiplatelet drugs**			1.000
No	74 (75.51%)	51 (76.12%)	
Yes	24 (24.49%)	16 (23.88%)	
**History of anticoagulant drugs**			1.000
No	90 (91.84%)	62 (92.54%)	
Yes	8 (8.16%)	5 (7.46%)	
No	42 (42.86%)	27 (40.30%)	
Yes	56 (57.14%)	40 (59.70%)	
**History of statin drugs**			1.000
No	72 (73.47%)	49 (73.13%)	
Yes	26 (26.53%)	18 (26.87%)	
**History of hypoglycemic drugs**			0.905
No	77 (78.57%)	54 (80.60%)	
Yes	21 (21.43%)	13 (19.40%)	

Continuous variables are expressed as Mean ± SD and categorical variables are expressed as No. (%).

**Table 6:** Analysis of laboratory test results with different functional outcome groups at 30 days after intravenous thrombolysis [mean ± SD/No. (%), *N* = 165].

**Variable**	**Good functional outcome (mRS 0–2, *N =* 98)**	**Poor functional outcome (mRS 3–6, *N =* 67)**	***P***
DNT (min)	69.38 ± 37.59	72.12 (40.81)	0.662
PLT (10^9^/L)	172.66 ± 55.41	184.85 ± 70.23	0.287
INR	1.23 ± 0.50	1.16 ± 0.24	0.351
Fg (g/L)	2.81 ± 1.44	3.08 ± 1.24	0.293
TG (mmol/L)	3.95 ± 1.14	3.94 ± 0.91	0.986
HDL (mmol/L)	1.23 ± 0.30	1.25 ± 0.30	0.720
LDL (mmol/L)	2.22 ± 0.88	2.20 ± 0.76	0.892
TyG	8.68 ± 0.29	8.85 ± 0.36	< 0.001

At 30 days after intravenous thrombolysis, 98 people were included in the good functional prognosis group and 67 people in the poor functional prognosis group. No significant differences were found in the baseline characteristics of patients between the two outcome groups (*P* > 0.05). These results are detailed in Table 5.

Table 5 analysis of people with different functional outcome [mean ± SD/No. (%), *N* = 165] at 30 days after intravenous thrombolysis. Continuous variables are reported as mean ± standard deviation (SD), whereas categorical variables are presented as counts (No.) (%).

There was no difference in DNT, PLT, INR, Fg, TG, HDL, and LDL between the two groups. The median TyG index in the good functional outcome group was 8.68 ± 0.29, and in the poor functional outcome group was 8.85 ± 0.36. Significant differences were found between the two groups (*P* < 0.001). Table 6 presents the findings.

### 3.4 Analysis of patients with different functional outcomes of the TyG index results at 90 days after intravenous thrombolysis

A normality test revealed that the HyG index data were skewed, resulting in the use of the Kruskal-Wallis test and Chi-square test (Table 7), one-way analysis of variance (ANOVA) (Table 8), and the median test for analysis.

**Table 7:** Analysis of baseline characteristics with different functional outcome groups at 90 days after intravenous thrombolysis.

**Variable**	**Good functional outcome (mRS 0–2, *N =* 94)**	**Poor functional outcome (mRS 3–6, *N =* 71)**	***P***
**Sex**			0.049
Male	58 (61.70%)	32 (45.07%)	
Female	36 (38.30%)	39 (54.93%)	
Age	69.39 ± 11.67	72.75 ± 12.57	0.082
First hospitalization SBP (mmHg)	149.57 ± 24.44	144.36 ± 23.61	0.194
First hospitalization DBP (mmHg)	83.36 ± 15.52	89.20 ± 19.78	0.056
**Smoking**			0.357
No	49 (52.13%)	43 (60.56%)	
Yes	45 (47.87%)	28 (39.44%)	
**Alcohol consumption**			0.040
No	59 (62.77%)	56 (78.87%)	
Yes	35 (37.23%)	15 (21.13%)	
**History of hypertension**			0.824
No	33 (35.11%)	27 (38.03%)	
Yes	61 (64.89%)	44 (61.97%)	
**History of diabetes**			0.530
No	69 (73.40%)	56 (78.87%)	
Yes	25 (26.60%)	15 (21.13%)	
History of dyslipidemia			0.735
No	75 (79.79%)	59 (83.10%)	
Yes	19 (20.21%)	12 (16.90%)	
**History of cerebral infarction**			0.850
No	79 (84.04%)	58 (81.69%)	
Yes	15 (15.96%)	13 (18.31%)	
History of CHD			0.800
No	81 (86.17%)	63 (88.73%)	
Yes	13 (13.83%)	8 (11.27%)	
**History of atrial fibrillation**			0.829
No	73 (77.66%)	57 (80.28%)	
Yes	21 (22.34%)	14 (19.72%)	
**History of antiplatelet drugs**			0.794
No	70 (74.47%)	55 (77.46%)	
Yes	24 (25.53%)	16 (22.54%)	
**History of anticoagulant drugs**			0.597
No	88 (93.62%)	64 (90.14%)	
Yes	6 (6.38%)	7 (9.86%)	
No	39 (41.49%)	30 (42.25%)	
Yes	55 (58.51%)	41 (57.75%)	
History of statin drugs			0.878
No	68 (72.34%)	53 (74.65%)	
Yes	26 (27.66%)	18 (25.35%)	
History of hypoglycemic drugs			0.960
No	74 (78.72%)	57 (80.28%)	
Yes	20 (21.28%)	14 (19.72%)	

Continuous variables are expressed as Mean ± SD and categorical variables are expressed as No. (%).

**Table 8:** Analysis of laboratory test results with different functional outcome groups at 90 days after intravenous thrombolysis [mean ± SD/No. (%), *N* = 165].

**Variable**	**Good functional outcome (mRS 0–2, *N =* 94)**	**Poor functional outcome (mRS 3–6, *N =* 71)**	
DNT (min)	69.80 ± 37.82	71.41 ± 40.39	0.795
PLT (10^9^/L)	172.17 ± 56.60	185.13 ± 69.04	0.256
INR	1.23 ± 0.51	1.17 ± 0.24	0.397
Fg (g/L)	2.79 ± 1.47	3.10 ± 1.20	0.229
TG (mmol/L)	3.94 ± 1.13	3.95 ± 0.95	0.921
HDL (mmol/L)	1.23 ± 0.31	1.25 ± 0.29	0.669
LDL (mmol/L)	2.21 ± 0.85	2.21 ± 0.81	0.976
TyG index	8.66 ± 0.28	8.87 ± 0.35	< 0.001

At 90 days after intravenous thrombolysis, 94 people were assigned to the good functional prognosis group and 71 people in the poor functional prognosis group. Baseline characteristics did not differ significantly between the two outcome groups (*P* > 0.05), except for sex (*P* = 0.049) and alcohol consumption (*P* = 0.040). The findings are presented in Table 7.

Table 7 analysis of people with different functional outcome [mean ± SD/No. (%), *N* = 165] at 90 days after intravenous thrombolysis. Continuous variables are presented as mean ± standard deviation (SD), whereas categorical variables are represented as No. (%).

There was no difference in DNT, PLT, INR, Fg, TG, HDL, and LDL between the two groups. The median TyG index in the good functional outcome group was 8.66 ± 0.28, and in the poor functional outcome group was 8.87 ± 0.3. There were notable differences between the two groups (*P* < 0.001). The results are displayed in Table 8.

### 3.5 Logistic regression analysis for modeling disease-related influencing factors

**Figure 2:**
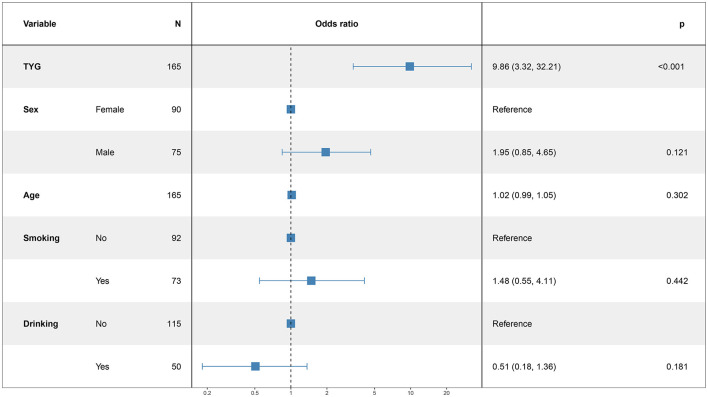
The multiple logistic regression model of 14 days after intravenous thrombolysis.

**Figure 3:**
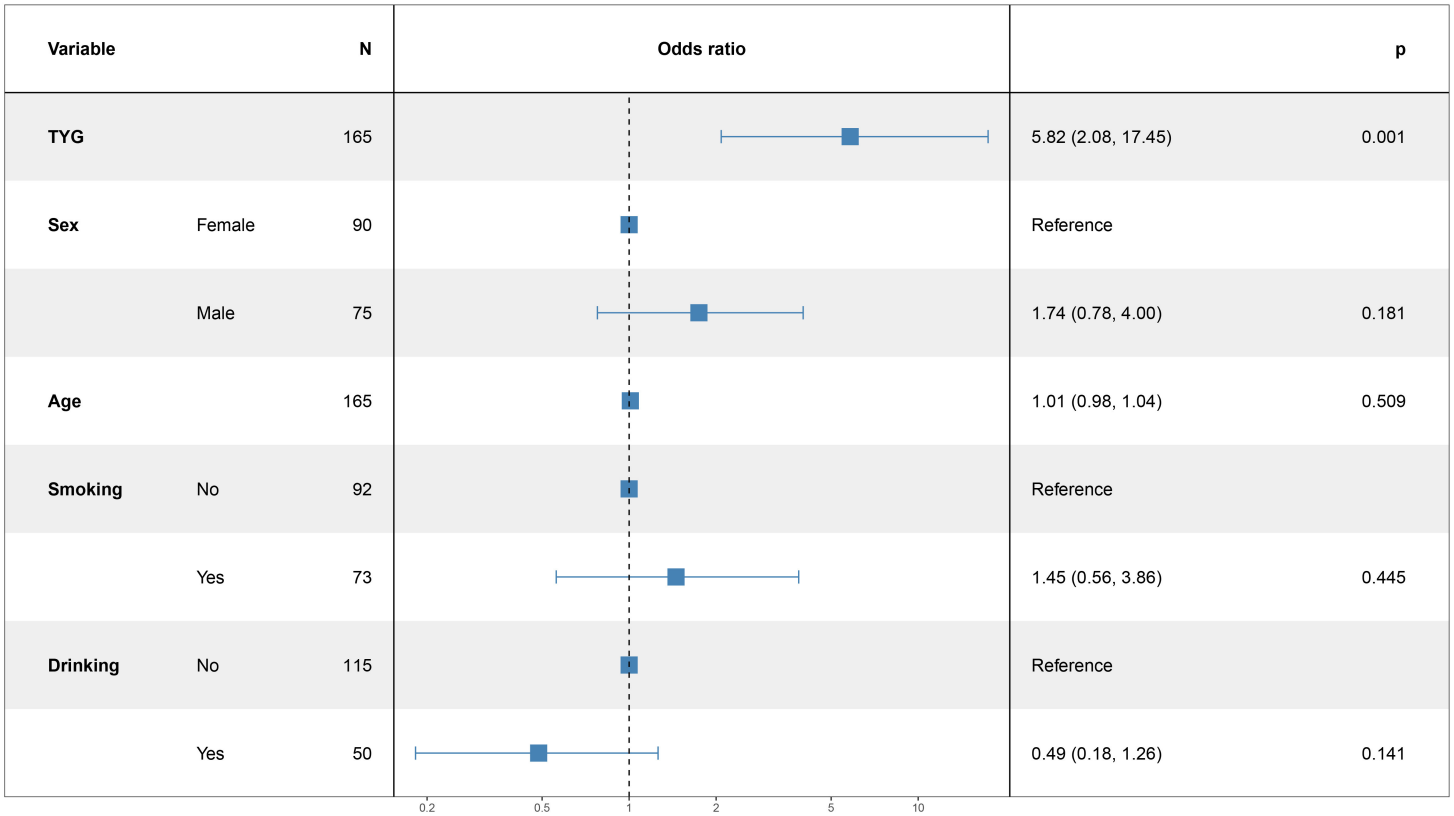
The multiple logistic regression model of 30 days after intravenous thrombolysis.

**Figure 4:**
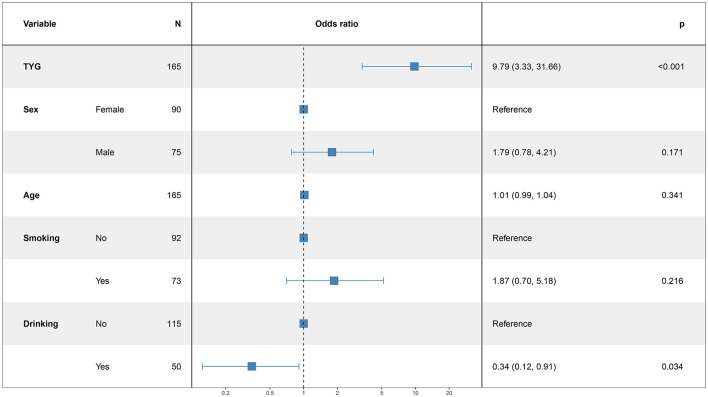
The multiple logistic regression model of 60 days after intravenous thrombolysis.

In this study, the patient's functional outcome status was treated as the dependent variable, with the mRS and baseline information used as independent variables to develop a logistic regression model. The final determinants included in the model are depicted in Figure 2 (14 mRS), Figure 3 (30 mRS), and Figure 4 (60 mRS). Calibration curves (Supplementary Figures S1–S3) demonstrated good predictive performance For each unit increase in the TyG index, patients with poor functional outcomes at 14, 30, and 90 days respectively changed to 9.86 (3.32, 32.21, *P* < 0.001), 5.82 (2.08, 17.45, *P* = 0.001), 9.79 (3.33, 31.66, *P* < 0.001), indicating that the higher the TyG index. The results also indicate that men, increasing age and smoking also lead to worse neurological outcomes in stroke patients. Although statistically not significant, the model results indicated that alcohol consumption did not serve as a major determinant of patients' functional outcomes. It has been indicated that alcohol consumption might contribute to the occurrence of functional impairments.

## 4 Discussion

Intravenous thrombolysis with alteplase remains the most effective treatment for acute ischemic stroke when administered within 4.5 h of symptom onset. Alteplase is a tissue plasminogen activator (t-PA) produced using recombinant DNA technology, and it acts similarly to the naturally occurring t-PA. It activates plasminogen bound to fibrin, thereby dissolving blood clots and restoring arterial patency (24, 25). However, some patients still experience poor outcomes following thrombolysis.

Patients with acute ischemic stroke often suffer from stress-induced elevated blood glucose and insulin resistance and these adverse complications often seriously affect the prognosis of patients (26). Foreign studies show that the acute ischemic stroke patients with the body often is in a state of stress, stimulate the hypothalamic-pituitary-adrenal axis and sympathetic excitation, cortisol, catecholamine and other stress hormones release, causing stress hyperglycemia and insulin resistance occurs (25), epidemiological investigation shows that the incidence of stress hyperglycemia in acute critical disease as high as 33.00%, the incidence of acute stroke is 30.00% (27). Blood glucose management is a crucial component in the clinical management of ischemic stroke. Insulins and hypoglycemic drugs are commonly used in clinical practice, but the effects are often poor, indicating that there may be various mechanisms to promote the clinical exploration of new therapeutic targets.

The TyG index is a recently proposed and easily calculable peripheral insulin resistance surrogate marker (15, 28). Abnormal triglyceride levels in the liver and muscle tissues are significant factors contributing to insulin resistance, indicating the biological feasibility of using the TyG index as a surrogate marker for insulin resistance. Insulin resistance is often linked to disturbances in both lipid and glucose metabolism (29).

Studies have reported that insulin resistance can occur not only peripherally, but also within neural cells. Central insulin resistance is linked to various diseases such as Alzheimer's disease, cardiovascular and cerebrovascular diseases, cognitive impairment and metabolic syndrome (30). However, methods for assessing central insulin resistance are limited and complex, restricting their clinical application. Central insulin resistance often accompanies peripheral hyperinsulinemia and peripheral insulin resistance (31). Consequently, research frequently focuses on evaluating peripheral insulin resistance markers to explore their relationship with related diseases.

The “gold standard” for assessing peripheral insulin resistance is time-consuming and labor-intensive, requiring insulin level measurements, which limits its feasibility in clinical practice, especially in primary care. In contrast, the TyG index, initially designed as a marker for chronic insulin resistance (IR) (15, 32), is both reliable and simple (28). Thus, the close association between the TyG index and early recurrent ischemic lesions (ERIL) may reflect several pathological conditions associated with chronic IR, such as metabolic diseases, subclinical inflammation, and endothelial dysfunction (33–35). One study (36) found that the TyG index was closely related to inflammatory markers and the TG/HDL ratio, another IR marker (37), suggesting that chronic IR may influence ERIL occurrence. Although the TG/HDL ratio was not significantly associated with ERIL, it showed a trend toward correlation. The TyG index may also reflect the combined effects of hyperglycemia and hypertriglyceridemia, known to worsen the acute prognosis of ischemic stroke (38, 39). Additionally, the TyG index may promote ischemic stroke progression and recurrence by reducing responsiveness to aspirin in patients receiving antiplatelet therapy (40).

Meta-analyses have shown that those with the highest TyG index are at a significantly greater risk of stroke. A high TyG index is associated with a heightened likelihood of cerebral infarction and non-fatal stroke (41). An elevated TyG index could act as an indicator of stroke risk, particularly ischemic stroke. In this study, the average TyG index for patients with acute ischemic stroke was 9.13 ± 0.62.

In patients who underwent intravenous thrombolysis within 4.5 h of symptom onset, an elevated TyG index was associated with a greater risk of early neurological deterioration and a reduced probability of early neurological improvement (42). Additionally, the study found that a higher TyG index correlated with poorer neurological outcomes at 14 days, 30 days, and 90 days post-stroke.

This study indicates that alcohol consumption is a protective factor for neurological recovery after cerebral infarction (*P* = 0.04). Previous research has shown that moderate ethanol consumption can increase insulin sensitivity reduce platelet aggregation and raise high-density lipoprotein cholesterol (HDL-C) levels. However, excessive alcohol consumption can lead to lactic acid accumulation, ketoacidosis and an increased risk of diabetes and lacunar infarctions. HDL-C is known to be a protective factor against cerebrovascular diseases. Numerous studies have demonstrated a dose-dependent positive correlation between alcohol consumption and HDL-C levels, with HDL-C increasing as alcohol intake rises. For example, daily ethanol intake of 30 g can raise HDL-C levels by 0.103 mmol/L (39.9 mg/dL); drinking once daily can increase HDL-C by 5%, and drinking 2–3 times a day can increase it by 10% (43). The exact mechanism by which ethanol increases HDL-C levels is not fully understood. However, some studies suggest that while alcohol has the potential to markedly elevate HDL-C levels. It does not necessarily reduce the risk of cerebrovascular diseases. Schutte et al. (44) described a similar situation, tracking 333,259 consumers of alcohol and 21,710 abstainers from the UK Biobank over a period of 6.9 years. They discovered that alcohol consumption was associated with a reduced risk of coronary artery disease (CAD) and cerebrovascular disease compared to non-drinking. In another analysis by Biddinger et al. (46), which included 371,463 individuals from the UK Biobank and employed Mendelian randomization, alcohol consumption was linked to elevated cardiovascular risks, including stroke (OR, 1.26; 95% CI, 1.04–1.54), heart failure (OR, 1.39; 95% CI, 1.08–1.78), hypertension (odds ratio [OR], 1.28; 95% CI, 1.18–1.39), acute coronary syndrome (OR, 1.38; 95% CI, 1.1–1.74), atrial fibrillation (OR, 1.24; 95% CI, 1.08–1.44), and CAD (OR, 1.38; 95% CI, 1.1–1.74). Lankester et al. (47) similarly identified this trend in their analysis of 337,484 individuals from the UK Biobank. Their findings indicated that each additional daily alcoholic drink was associated with an increased risk of hemorrhagic stroke (OR, 2.25; 95% CI, 1.41–3.6), a rise in systolic blood pressure (β = 2.65 mm Hg; 95% CI, 1.4–3.89), and a higher likelihood of atrial fibrillation (OR, 1.26; 95% CI, 1.07–1.48) (45). Further research is needed to precisely quantify the impact of alcohol consumption.

The TyG index, as a novel alternative marker for insulin resistance, is simple, reliable, and readily accessible. This study is the first to explore the relationship between the TyG index and acute ischemic stroke, and it suggests that the TyG index may serve as a predictive marker for acute ischemic stroke. We plan future studies to assess stroke prognosis in acute ischemic stroke patients based on the TyG index, implement early interventions targeting fasting glucose and triglyceride levels, and reduce the TyG index to improve neurological outcomes. However, there are certain limitations. First, the study is retrospective, and the small sample size may introduce bias into the results. Additionally, the relationship between other TyG-derived indices and acute ischemic stroke was not analyzed. Future research should involve prospective, multicenter, large cohort studies to more comprehensively address these issues.

## 5 Conclusion

This study found a positive correlation between the TyG index and acute ischemic stroke. A higher TyG index, reflecting greater insulin resistance, was associated with worse neurological prognosis in these patients. Early intervention targeting insulin resistance may improve clinical outcomes in ischemic stroke. Further research is needed to identify additional factors that affect neurological recovery.

## Data Availability

The original contributions presented in the study are included in the article/Supplementary material, further inquiries can be directed to the corresponding author.
