# Supplementary material for: Analyzing the correlation between acute ischemic stroke and triglyceride-glucose index based on ordered logistic regression
Source: Front Neurol. 2025 Feb 5;16:1500572. doi: 10.3389/fneur.2025.1500572 (PMC11835692; doi:10.3389/fneur.2025.1500572)
Supplement: Supplementary file 1 [file Data_Sheet_1.docx]

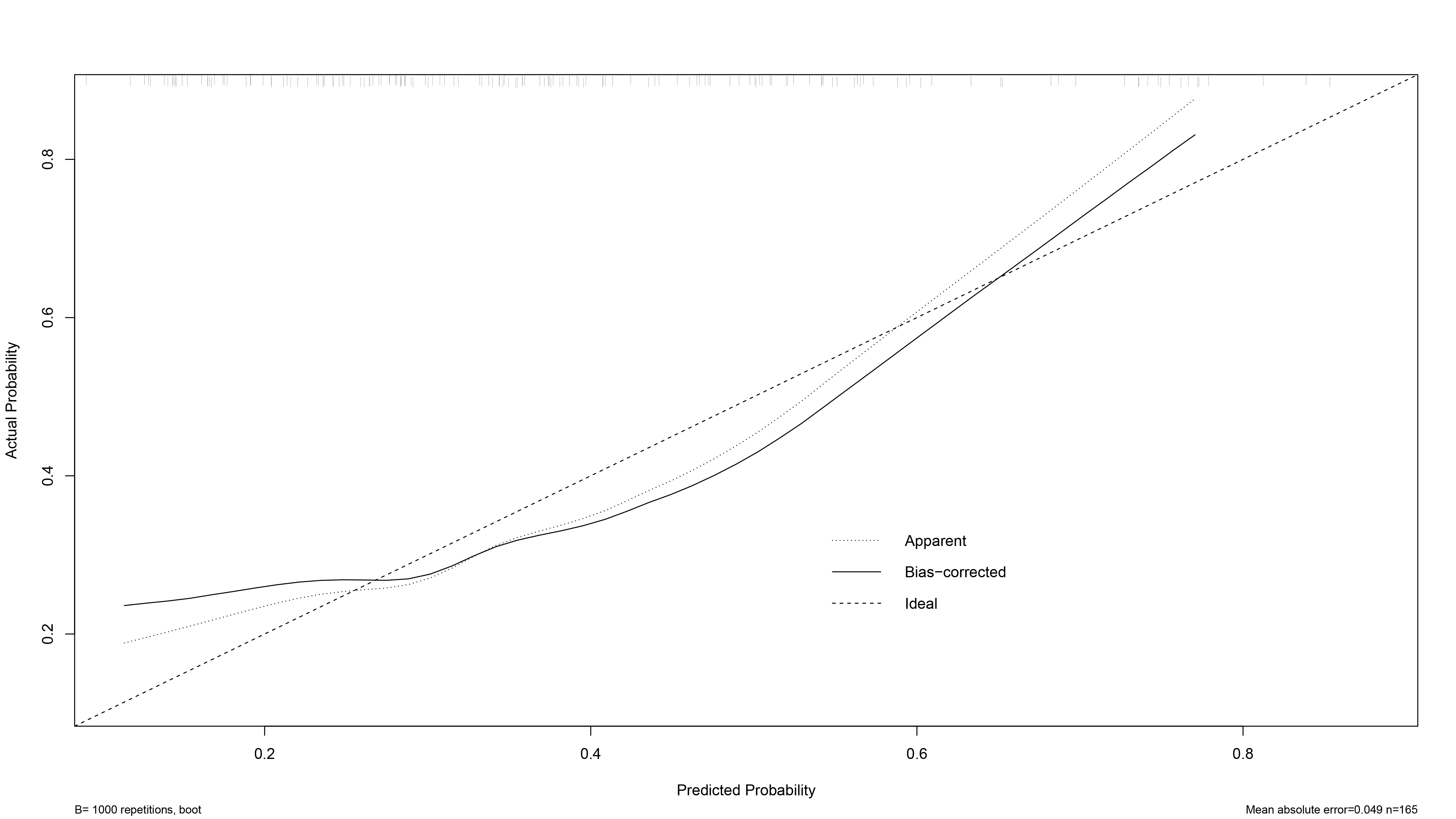


FigureS1 Calibration curves of the multiple logistic regression model of 14 mRS


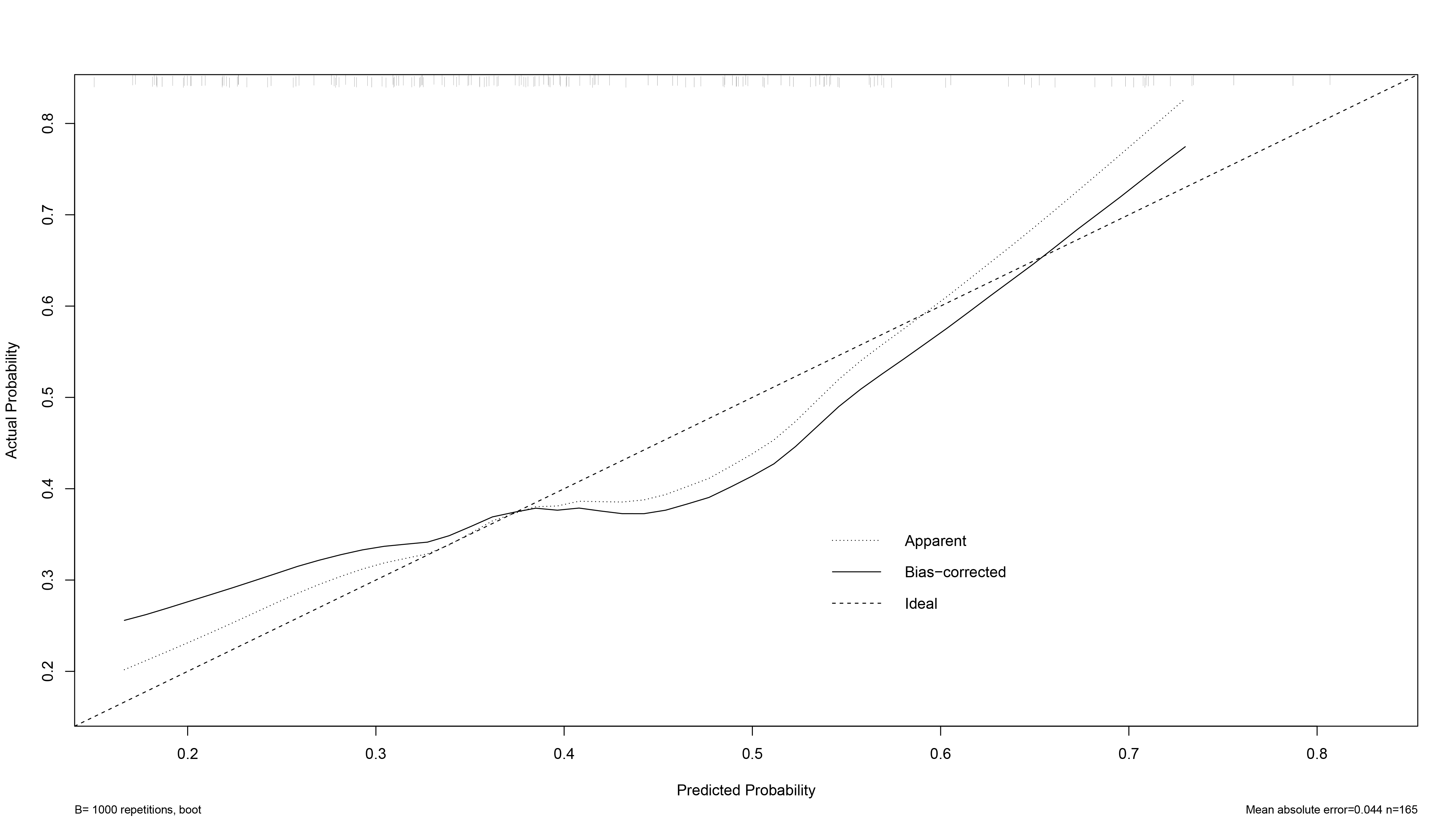


FigureS2 Calibration curves of the multiple logistic regression model of 30 mRS


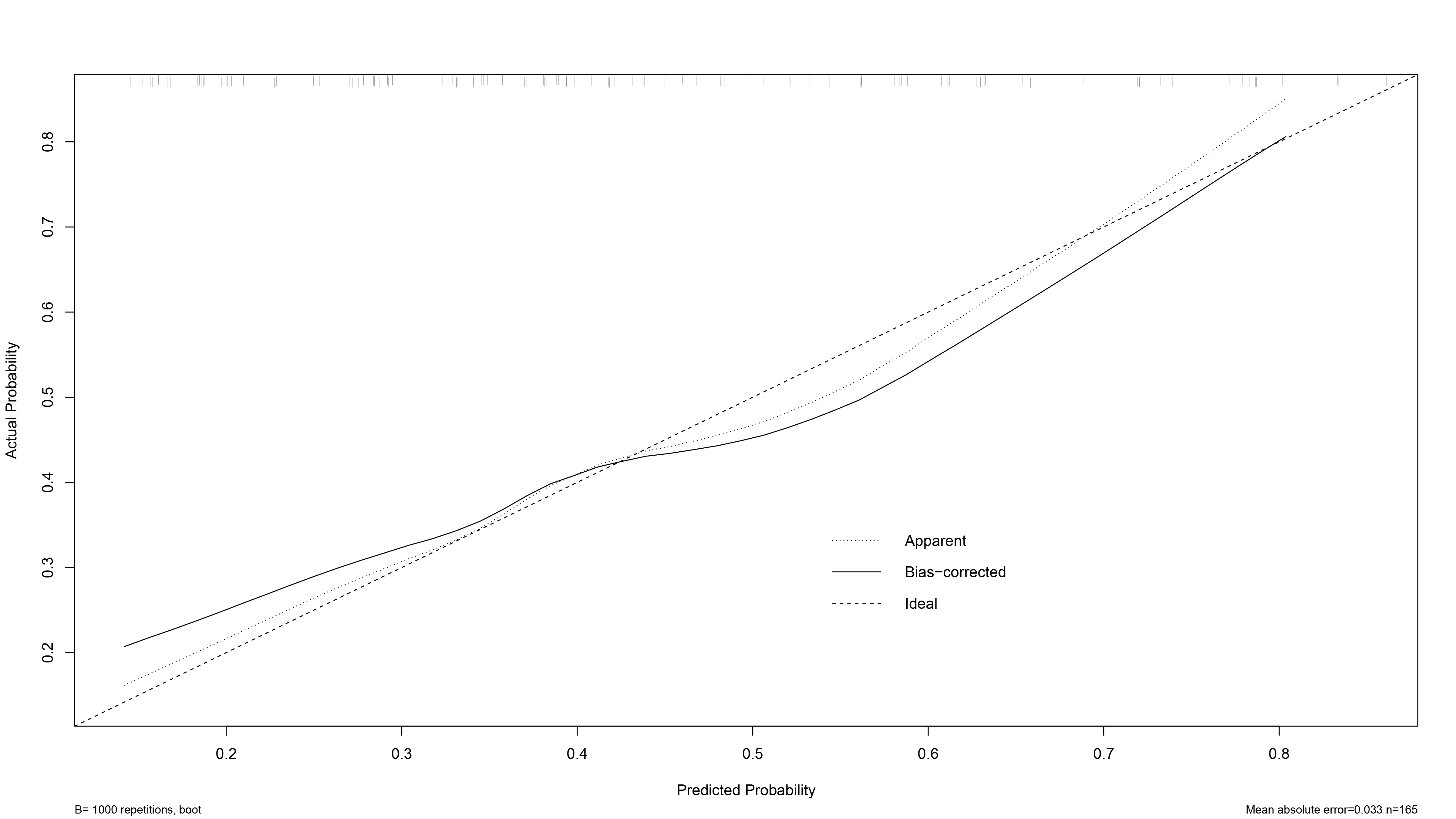


FigureS3 Calibration curves of the multiple logistic regression model of 90 mRS
